# Management of laryngopharyngeal reflux in Brazil: a national survey

**DOI:** 10.1016/j.bjorl.2020.11.017

**Published:** 2020-12-29

**Authors:** Jerome R. Lechien, Paulo S. Perazzo, Fabio P. Ceccon, Claudia A. Eckley, Karen de Carvalho Lopes, Rebecca Maunsell, Melissa A.G. Avelino, Lee M. Akst, Geraldo D. Sant’Anna, Rui Imamura

**Affiliations:** a Young-Otolaryngologists of the International Federations of Otorhinolaryngological Societies (YO-IFOS), Laryngopharyngeal Reflux Study Group, Paris, France; b University of Mons (UMons), UMONS Research Institute for Health Sciences and Technology, Faculty of Medicine, Department of Human Anatomy and Experimental Oncology, Mons, Belgium; c Université Versailles Saint-Quentin-en-Yvelines (Paris Saclay University), School of Medicine, UFR Simone Veil, Foch Hospital, Department of Otorhinolaryngology and Head and Neck Surgery, Paris, France; d Université Libre de Bruxelles, School of Medicine, CHU Saint-Pierre, CHU de Bruxelles, Department of Otorhinolaryngology and Head and Neck Surgery, Brussels, Belgium; e Universidade Estadual da Bahia, Faculdade de Ciências Médicas, Escola de Medicina, Departamento de Otorrinolaringologia, Salvador, BA, Brazil; f Universidade Federal de São Paulo (UNIFESP), Departamento de Otorrinolaringologia e Cirurgia de Cabeça e Pescoço, São Paulo, SP, Brazil; g Fleury Medicina e Saúde Laboratórios Diagnósticos, Departamento de Otorrinolaringologia e Cirurgia de Cabeça e Pescoço, São Paulo, SP, Brazil; h Universidade Federal de São Paulo (UNIFESP), Escola Paulista de Medicina (EPM), Disciplina de Otologia e Otoneurologia, São Paulo, SP, Brazil; i Universidade Estadual de Campinas (UNICAMP), Faculdade de Ciências Médicas, Departamento de Otorrinolaringologia e Cirurgia de Cabeça e Pescoço, Campinas, SP, Brazil; j Universidade Federal de Goiás (UFG) Anápolis, Goiás, Brazil; k Johns Hopkins Hospital, Department of Otolaryngology-Head Neck Surgery, Baltimore, United States; l Universidade Federal de Ciências da Saúde de Porto Alegre (UFCSPA), Disciplina de Otorrinolaringologia, Porto Alegre, RS, Brazil; m Universidade de São Paulo, Faculdade de Medicina, Hospital das Clínicas, Departamento de Otorrinolaringologia, São Paulo, SP, Brazil

**Keywords:** Disease management, Awareness, Gastroesophageal reflux, laryngitis, Globus sensation

## Abstract

**Introduction:**

Studies assessing the management of laryngopharyngeal reflux by otolaryngologists have reported an important heterogeneity regarding the definition, diagnosis, and treatment, which leads to discrepancies in the management of the patient. Information about the current knowledge and practices of Brazilian otolaryngologists in laryngopharyngeal reflux is lacking.

**Objective:**

To investigate the trends in management of laryngopharyngeal reflux disease among Brazilian otolaryngologists.

**Methods:**

A survey was sent by email to the members of the Brazilian Association of Otolaryngology-Head Neck Surgery. This survey has initially been conducted by the laryngopharyngeal reflux study group of young otolaryngologists of the *International Federation of Otolaryngological Societies*.

**Results:**

According to the survey responders, the prevalence of laryngopharyngeal reflux was estimated to be 26.8% of patients consulting in otolaryngology and the most common symptoms were globus sensation, throat clearing, cough and stomach acid reflux. Nasal obstruction, Eustachian tube dysfunction, acute and chronic otitis media, vocal fold nodules and hemorrhage were considered not associated with laryngopharyngeal reflux by the majority of responders. About 2/3 of Brazilian otolaryngologists based the diagnosis of laryngopharyngeal reflux on the assessment of both symptoms and findings and a positive response to empiric therapeutic trials. Proton pump inhibitor utilized once or twice daily, was the most commonly used therapeutic scheme. Only 21.4% of Brazilian otolaryngologists have heard about nonacid and mixed laryngopharyngeal reflux and the awareness about the usefulness of multichannel intraluminal impedance pH monitoring (MII-pH) was minimal; 30.5% of responders did not consider themselves as well-informed about laryngopharyngeal reflux.

**Conclusion:**

Although the laryngopharyngeal reflux-related symptoms, main diagnostic and treatment approaches referred by Brazilian otolaryngologists are consistent with the literature, the survey identified some limitations, such as the insufficient awareness of the role of laryngopharyngeal reflux in many otolaryngological conditions and of the possibility of non-acid or mixed reflux in refractory cases. Future studies are needed to establish international recommendations for the management of laryngopharyngeal reflux disease.

## Introduction

Laryngopharyngeal reflux (LPR) is an inflammatory condition of the upper aerodigestive tract tissues related to direct and indirect effect of gastroduodenal content reflux, which induces morphological changes in the upper aerodigestive tract.^1^ The prevalence of LPR symptoms ranges from 10% to 35% of patients visiting Otolaryngology-Head & Neck Surgery Departments world-wide.^2^, ^3^ In Brazil, the otolaryngologists are front-line for patients with LPR-related symptoms and often have to manage both the diagnosis and the treatment of these patients. Recent European and Asian studies assessing the management of LPR by otolaryngologists have been conducted.^4^, ^5^ Overall, the results reported an important heterogeneity between otolaryngologists about the definition, diagnosis and treatment of LPR.^4^, ^5^ The results of these surveys highlight the current controversies in this area, which lead to discrepancies in the management of the patient. The current knowledge and practices of Brazilian otolaryngologists and laryngologists have never been evaluated.

The aim of this study is to investigate the current practices in management of LPR among Brazilian otolaryngologists.

## Methods

The questions of the survey have been written by the members of the LPR study group of young otolaryngologists of the International Federation of Otolaryngological Societies (YO-IFOS). The final version of the survey consisted of 21 questions divided into 5 sections: definition and epidemiology (3); clinical presentation (4); diagnostic approach (3); treatment (10) and skills (1). Through this survey presentation, the members of the LPR study group had to target all controversial LPR topics.

The survey was created with Survey Monkey (San Mateo, California, USA) through a system that ensured that each participant could complete the survey only once. The survey content was developed in iterative fashion, with drafts revised by three certified otolaryngologists and one family physician.

The survey was emailed once to members of the Associação Brasileira de Otorrinolaringologia e Cirurgia Cérvico-Facial.

The study protocol was approved by the Institutional Review Board of CHU de Bruxelles, Site Saint-Pierre (ref. LPR-122019). Responses were collated anonymously in an excel file. Statistical Package for the Social Sciences for Windows (SPSS version 22,0; IBM Corp, Armonk, NY, USA) was used to perform the statistical analyses. A level of *p* < 0.05 was used to determine statistical significance.

## Results

### Sample characteristics

A total of 5406 Otolaryngologists in Brazil received the survey by email. Of these, 220 completed the questionnaire (response rate: 4.1%). Four otolaryngologists did not complete all questions of the survey, and, therefore, were excluded. Otolaryngologists had 17.94 ± 10.68 years of practice as certified otolaryngologists (range: 1–44). The age of responders was roughly calculated by adding 25 years to the reported number of years in practice. The distribution of age according to this criteria was: <30 years (6.4%), 30–39 years (35.6%), 40–49 years (32.9%), 50–59 years (14.6%), 60+ years (10.5%). The majority of otolaryngologists have more than one subspecialty: 153 general otolaryngologists; 49 laryngologists; 16 head and neck surgeons; 39 otologists; 46 rhinologists and 38 pediatric otolaryngologists.

### Laryngopharyngeal reflux definition & prevalence

The mean prevalence of LPR was estimated to be 26.75 ± 15.52% of patients consulting in otolaryngology. 65.8% of otolaryngologists thought that LPR and gastroesophageal reflux disease (GERD) are clinically different diseases sharing some common pathophysiological mechanisms whereas 21.9% considered both LPR and GERD as the same disease. LPR and GERD were considered as two different diseases without common pathophysiological mechanisms by 11.4% of responders; 0.9% of otolaryngologists did not know.

### Laryngopharyngeal reflux involvement in otolaryngological conditions

**Figure 1:**
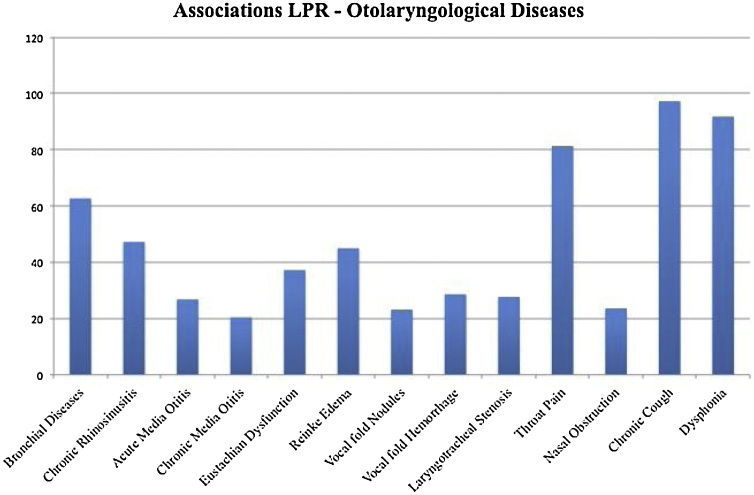
Association between laryngopharyngeal reflux and some ear, nose, and throat conditions. The ordinate axis corresponds to percentage of Brazilian otolaryngologists who think that there is an association with the condition.

The otolaryngological conditions that were considered as the most frequently associated with LPR were: chronic cough (97.3%); dysphonia (91%) and bronchial diseases (62.7%). The majority of otolaryngologists did not consider LPR as involved in the development of nasal obstruction, vocal fold nodules, Reinke edema, vocal fold hemorrhage, chronic rhinosinusitis, Eustachian dysfunction laryngotracheal stenosis and chronic and acute media otitis (Fig. 1).

### Laryngopharyngeal reflux clinical presentation

The symptoms and findings thought highly related to LPR are reported in Tables 1 and 2 . Otolaryngologists consider globus sensation, objective acid reflux and cough after lying down/eating as the symptoms that are the most frequently associated with LPR. Interviewed otolaryngologists consider the prevalence of heartburn in the LPR population as 10%–20% (19.5%), 20%–30% (29.0%), 30%–40% (20.7%), 40%–50% (14.1%), and more than 50% (16.6%) of cases. They consider laryngeal/arytenoid erythema, posterior commissure granulation and posterior commissure edema as the findings that are the most frequently associated with LPR. 23% of Brazilian otolaryngologists used patient reported outcome questionnaires for the diagnostic or the followup of LPR patients.

**Table 1:** Symptoms in terms of their association with laryngopharyngeal reflux.

	1	2	3	4	5
Heartburn	31.73	14.42	36.54	12.02	5.29
Stomach acid coming up/regurgitation	43.64	15.45	31.36	7.27	2.27
Troublesome cough	32.11	43.58	22.02	2.29	0.00
Cough after lying down/after meal	44.29	36.07	13.24	5.94	0.46
Globus sensation	65.60	22.94	8.72	2.75	0.00
Hoarseness/voice disorder	18.18	33.18	41.82	5.91	0.91
Throat pain	18.26	25.57	41.55	10.96	3.65
Odynophagia	10.50	21.92	41.55	21.46	4.57
Dysphagia	6.39	22.37	39.73	26.94	4.57
Chest pain	0.91	8.18	37.27	39.09	14.55
Throat sticky mucus or postnasal drip	25.91	30.91	33.64	8.64	0.91
Throat clearing	37.16	33.94	23.39	4.59	0.92
Tongue burning	6.39	14.16	44.75	26.48	8.22
Halitosis	9.13	19.18	37.90	26.03	7.76
Breathing difficulties	1.82	7.27	39.55	33.18	18.18

Please rate each of the following symptoms in terms of their association with laryngopharyngeal reflux: 1, highly related; 3, somewhat related; 5, not related. The values are percentages.

**Table 2:** Findings in terms of their association with laryngopharyngeal reflux.

	1	2	3	4	5
Laryngeal/arytenoid erythema	62.56	26.94	8.68	1.37	0.46
Hypopharyngeal and/or oropharyngeal erythema	22.94	33.49	35.78	7.34	0.46
Anterior tonsillar pillar erythema	3.65	13.70	43.84	20.09	18.72
Vocal fold erythema	12.79	32.42	44.29	9.59	0.91
Subglottic erythema	5.96	19.72	38.53	24.77	11.01
Subglottic edema	9.68	18.43	35.48	23.04	13.36
Posterior commissure edema	58.64	30.91	7.27	2.73	0.45
Posterior commissure inflammatory granulations	40.91	28.64	15.91	13.18	1.36
Post cricoid edema	33.18	34.09	24.55	5.45	2.73
Hypo-oropharyngeal wall edema	13.64	26.36	39.09	16.82	4.09
Vocal fold edema	10.00	25.91	47.27	15.91	0.91
Laryngeal ventricular edema	5.02	23.29	47.03	19.63	5.02
Tonsil pillars edema	2.74	7.76	28.77	37.90	22.83
Vocal fold lesions (i.e. nodules, polyps, leukoplakia, ulceration, granuloma)	8.60	23.08	47.51	19.46	1.36
Endolaryngeal sticky mucus	11.36	34.55	36.36	13.64	4.09
Uvula erythema/edema	5.50	14.22	31.19	38.07	11.01
Coated tongue	3.18	8.64	42.27	33.64	12.27
Lingual tonsil hypertrophy	8.33	21.30	35.19	23.15	12.04

Please rate each of the following findings in terms of their association with laryngopharyngeal reflux: 1, highly related; 3, somewhat related; 5, not related. The values are percentages.

### Diagnostic methods

65.9% of Brazilian otolaryngologists based the LPR diagnosis on the assessment of both symptoms and findings and a positive response to an empirical therapeutic trial. Note that 52.7% of all responders address the patient to the department of gastroenterology. Only 5.9% of otolaryngologists are aware about the use of multichannel intraluminal impedance pH monitoring (MII-pH). The most important barriers for the use of MII-pH were: the patient’s tolerance (67.3%); the cost of the technique (49.5%); the patient’s inconvenience (40.9%); the lack of skills for interpreting the results (36.8%); the lack of usefulness of the examination for the diagnosis (35.5%); the lack of knowledge about the indications (34.1%); the lack of knowledge about the MII-pH (24.5%) and the lack of time for learning the bases of the approach (21.4%).

The role of gastrointestinal (GI) endoscopy is not well defined among the Brazilian population of otolaryngologists according to the results of the survey. The main indications for performing GI endoscopy were: LPR with refractory symptoms (64.5%); before the use of long-term PPI therapy (41.8%); all patients with LPR symptoms (35.9%) and elderly patients (38.6%). Note that 2.3% of responders thought that GI endoscopy is not important in the management of LPR.

### Laryngopharyngeal reflux treatment

**Figure 2:**
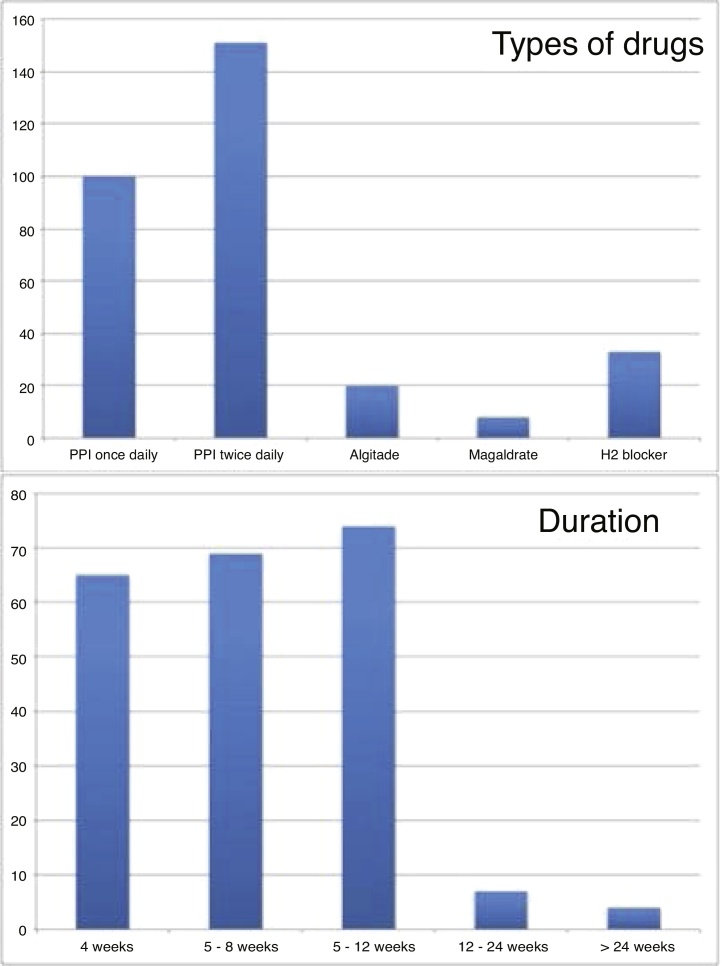
The therapeutic habits of Brazilian otolaryngologists. The duration and the types of medications used are described in this figure. 48.4% and 32.1% of otolaryngologist used twice and once daily PPI, respectively (10.6% use H2 blocker, 6.4% alginate and 2.6% magaldrate). (H2 blocker, Antihistamine; PPI, Proton Pump Inhibitor).

The various therapeutic approaches used by physicians are summarized in Fig. 2. 98.2% of Brazilian otolaryngologist’s advise diet and behavioral changes to their patients. The therapeutic scheme that is the most used by Brazilian otolaryngologists is: proton pump inhibitor (PPI) twice daily and PPI once daily. Alginate, magaldrate and anti-H2 blockers are less used than PPI as single therapy. The duration of treatment varied among responders: the majority considered duration of 8–12 weeks of treatment (Fig. 2). 52.3% of responders assess the treatment efficacy through symptom changes, whereas 47.3% consider changes of both symptoms and findings; 0.5% of responders only consider finding changes as therapeutic outcome. The Brazilian otolaryngologists consider that 73.4% of LPR patients respond to treatment.

### Management of resistant patients

**Figure 3:**
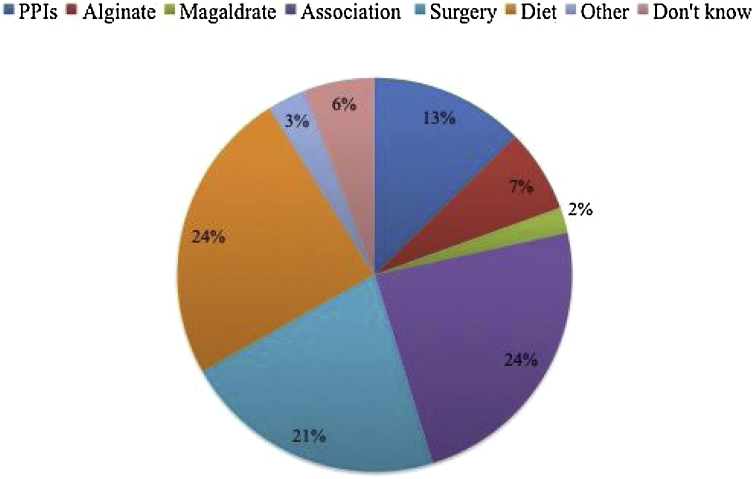
Treatment pattern of resistant patients (PPI, Proton Pump Inhibitor).

The majority of Brazilian otolaryngologists think that the main cause of resistance to treatment is the poor diet of patients (65.8%). The following other causes are thought by responders: severity of LPR (14.2%); lack of compliance to treatment (6.8%); and biliary reflux (5.5%); 7.8% of responders do not know the cause of resistance. Note that only 21.4% of Brazilian otolaryngologists have heard about nonacid and mixed LPR. The management of LPR patients who are resistant to treatment differs among responders. 45% of responders address the patient to the GI department; 34.1% use other medications than those initially prescribed; 12.7% prescribe long-term PPI therapy (although lack of improvement); 7.3% only give diet and behavioral advices and 0.9% address the patient to the surgery department for fundoplicature. The medications that are the most used for resistant patients are reported in Fig. 3. More than 50% of Brazilian otolaryngologists prescribed strict diet (24%), association of drugs (24%) or surgery (21%) for the resistant patients.

### Skills

The majority of Brazilian otolaryngologists consider themselves well informed about LPR (51.8%), whereas 30.5% think the opposite and 17.7% do not know.

## Discussion

The number of studies devoted to LPR have gradually increased over the past two decades.^6^, ^7^ However, management of LPR is still controversial, especially concerning the diagnostic approach and treatment of these patients; additionally, many physicians are not aware of the prevalence of this condition. This survey is the first study of this size designed to examine the current management of LPR by otolaryngologists in Brazil.

The prevalence of LPR according to Brazilian otolaryngologists (26.8% ± 15.5%) is consistent with the literature.^2^, ^3^, ^6^ According to the majority of responders, LPR is not associated with nasal obstruction, Eustachian dysfunction, acute and chronic otitis media, laryngotracheal stenosis, and the following benign lesions of the vocal folds: nodules and Reinke’s edema. In a recent European survey, our group found that European otolaryngologists also consider that LPR is not associated with Reinke’s edema and vocal fold nodules but, contrarily to Brazilian otolaryngologists, they think that LPR could be associated with nasal obstruction.^4^

In Asia, otolaryngologists recognize an association between Reinke’s edema and LPR but not with nasal obstruction, chronic and acute otitis media and laryngotracheal stenosis.^5^ In the literature, clinical and experimental studies suggested that LPR could be associated with laryngotracheal stenosis,^8^, ^9^, ^10^ acute otitis media^11^, ^12^ and Eustachian dysfunction.^12^ The role of LPR in the development of benign lesions of the vocal folds, especially nodules, Reinke’s edema and polyps, is also highly probable.^13^ In order for these chronic conditions to be adequately treated, the possibility that anti-reflux drugs might be a helpful adjunct in care needs to be considered.

The most common LPR-related symptoms in the opinion of Brazilian responders are globus sensation, throat clearing, cough after lying down and stomach acid reflux. In fact, globus sensation, throat clearing, hoarseness, excess throat mucus and postnasal drip are the most prevalent symptoms associated with LPR, according to a recent systematic review.^1^ Throat pain was referred by less than 20% of responders in our survey. The prevalence of throat pain is poorly reported in studies of LPR, perhaps due to the lack of consideration of this complain in well-used patient-reported outcome questionnaires, such as reflux symptom index.^14^ However, throat pain might be present in 68.5% of LPR patients, making it potentially one of the most prevalent complaints of patients with confirmed LPR.^15^, ^16^ The most prevalent symptoms regarding European and Asian otolaryngologists are quite similar.^4^, ^5^

In the current study, 2/3 of Brazilian otolaryngologists based the diagnosis of LPR on the assessment of both symptoms and findings and a positive response to empiric therapeutic trials. This diagnostic approach is used widely around the world^17^, ^18^ despite its limitations relative to false positives caused by the fluctuating nature of vague complaints such as throat clearing, globus pharyngeus, etc. which may resolve on their own—the response to placebo is often as high as it is to PPI.^1^, ^6^ In addition, because Brazil is a big country with huge different realities, it could justify the empiric treatment instead the regular use of the exams for diagnosis.

In fact, the most validated diagnostic approach for LPR is hypopharyngeal-esophageal MII-pH.^19^ However, as for European and Asian studies,^4^, ^5^ our study shows that the great majority of Brazilian otolaryngologists do not use MII-pH. The main barriers to its use are patient inconvenience and lack of tolerance, cost of the technique, lack of usefulness of the examination for the diagnosis, and lack of knowledge about the MII-pH and its indications. Many physicians recognize that they cannot interpret the results of MII-pH and are not aware of the prevalence and importance of nonacid and mixed LPR. The limited knowledge about MII-pH and other pH study examinations may explain why 52.7% of responders refer patients to gastroenterologists at baseline. This point may be related to the lack of availability of MII-pH in many South America centers; the insurance companies push for pHmetry, which is cheaper. Moreover, we do not address how easy is the assessment of diagnostic exams among Brazilian otolaryngologists, as dual-probe pH testing and esophageal manometry, for example. This could be an explanation for using symptoms and laryngeal examination for diagnosing LPR. F

Regarding treatment, once or twice daily PPIs are still the most used therapeutic regimens in Brazil. As for Asian and European otolaryngologists, the majority of Brazilian otolaryngologists advise diet and behavioral changes to their patients. The duration of empirical treatment prescribed by most of responders was up to 3-months, which differs from Asian and European studies where the duration of medication is shorter (1 month).^4^, ^5^ Some studies furthermore suggested that LPR findings may require more time to improve.^20^, ^21^

The estimated success rate of treatment is 73% according to responders, which is higher than the success rate reported in the literature.^22^

Brazilian otolaryngologists thought that the most frequent cause of resistance to treatment is the poor diet of patients (65.8%), followed by severity of LPR (14.2%) and lack of compliance to treatment (6.8%). Poor dietary habits may effectively play an important role in the lack of therapeutic response with regard to some studies reporting diet and lifestyle to be important factors in management of LPR.^23^, ^24^ Compliance to treatment is a real concern, as a recent study of Pisegna et al. demonstrated that 62.7% of individuals did not adequately take their medication.^25^ The respect of diet and medication intake is an important fact that should be considered in all resistant patients.

This study has some limitations. Participation bias may mean that the otolaryngologists who did respond felt themselves more informed about LPR than their non-respondent peers, but only 22.3% have laryngology as main specialty. Also, surveys based on digital recruitment are particularly prone to sampling bias, with a tendency of higher yields among younger physicians. However, the age distribution of our sample was very similar to that of a recent census of Brazilian otolaryngologists.^26^ In the future, as for U.S. studies,^27^ it will be interesting to conduct similar study in order the compare longitudinal trends in practice changes. On the other hand, this was the first study to deeply evaluate the management of LPR by Brazilian otolaryngologists. Results suggest that efforts must be taken to improve the knowledge of Brazilian otolaryngologists about the current evidence on the diagnosis and treatment of LPR. Establishing international recommendations might be the first step in improving physician practice.

## Conclusion

This study highlights the lack of awareness of Brazilian otolaryngologists about LPR. Many points suggested that the LPR is still poorly understood, which may impact the management of the disease. Because LPR is a prevalent and common disorder, it is important to improve the understanding or pathophysiology, treatments, and care knowledge of LPR by otolaryngologists and to conduct future studies that will resolve many unanswered questions.

## Conflicts of interest

The authors declare no conflicts of interest.
